# Mitochondrial Respiration in Insulin-Producing β-Cells: General Characteristics and Adaptive Effects of Hypoxia

**DOI:** 10.1371/journal.pone.0138558

**Published:** 2015-09-24

**Authors:** Ingrid K. Hals, Simon Gustafson Bruerberg, Zuheng Ma, Hanne Scholz, Anneli Björklund, Valdemar Grill

**Affiliations:** 1 Department of Cancer Research and Molecular Medicine, Faculty of Medicine, Norwegian University of Science and Technology (NTNU), Trondheim, Norway; 2 Department of Molecular Medicine and Surgery, Karolinska Institutet, Stockholm, Sweden; 3 Department of Transplantation Medicine and Institute for Surgical Research, Oslo University Hospital, Oslo, Norway; 4 Department of Endocrinology, St Olav University Hospital, Trondheim, Norway; Broad Institute of Harvard and MIT, UNITED STATES

## Abstract

**Objective:**

To provide novel insights on mitochondrial respiration in β-cells and the adaptive effects of hypoxia.

**Methods and Design:**

Insulin-producing INS-1 832/13 cells were exposed to 18 hours of hypoxia followed by 20–22 hours re-oxygenation. Mitochondrial respiration was measured by high-resolution respirometry in both intact and permeabilized cells, in the latter after establishing three functional substrate-uncoupler-inhibitor titration (SUIT) protocols. Concomitant measurements included proteins of mitochondrial complexes (Western blotting), ATP and insulin secretion.

**Results:**

Intact cells exhibited a high degree of intrinsic uncoupling, comprising about 50% of oxygen consumption in the basal respiratory state. Hypoxia followed by re-oxygenation increased maximal overall respiration. Exploratory experiments in peremabilized cells could not show induction of respiration by malate or pyruvate as reducing substrates, thus glutamate and succinate were used as mitochondrial substrates in SUIT protocols. Permeabilized cells displayed a high capacity for oxidative phosphorylation for both complex I- and II-linked substrates in relation to maximum capacity of electron transfer. Previous hypoxia decreased phosphorylation control of complex I-linked respiration, but not in complex II-linked respiration. Coupling control ratios showed increased coupling efficiency for both complex I- and II-linked substrates in hypoxia-exposed cells. Respiratory rates overall were increased. Also previous hypoxia increased proteins of mitochondrial complexes I and II (Western blotting) in INS-1 cells as well as in rat and human islets. Mitochondrial effects were accompanied by unchanged levels of ATP, increased basal and preserved glucose-induced insulin secretion.

**Conclusions:**

Exposure of INS-1 832/13 cells to hypoxia, followed by a re-oxygenation period increases substrate-stimulated respiratory capacity and coupling efficiency. Such effects are accompanied by up-regulation of mitochondrial complexes also in pancreatic islets, highlighting adaptive capacities of possible importance in an islet transplantation setting. Results also indicate idiosyncrasies of β-cells that do not respire in response to a standard inclusion of malate in SUIT protocols.

## Introduction

The pancreatic β-cell is metabolically very active and therefore highly dependent on oxygen supply [1, 2]. Its elevated oxygen demand is a prerequisite for the mitochondrial metabolism of glucose which provides for insulin biosynthesis, as well as the signaling pathway for insulin secretion, the β-cell’s main function [3]. Reduced oxygen tension, hypoxia, in β-cells is implicated in several pathological conditions (including hyperglycemia) [4], and may be a major factor behind non-optimal results of pancreatic islet transplantation [5].

Changes in mitochondrial function are expected to occur in hypoxia-induced loss of function. However, it is not fully understood how reduced oxygen availability affects mitochondrial function in β-cells. In fact, little research has been done on the effect of hypoxia on respiratory capacities and mitochondrial coupling states in β-cells, especially with regard to involvement of individual mitochondrial complexes. More knowledge on how hypoxia and re-oxygenation affects respiration and mitochondrial markers would provide insights that could be valuable for improving β-cell survival and function after transplantation. Of special interest are adaptive processes. Adaptation to a limited degree of hypoxia (often associated with activation of HIF1-alpha) [2] is documented in many cell types [6] and could, if augmented in β-cells, be used as pre-conditioning to alleviate the negative impact of hypoxia on β-cells after transplantation. Indeed some evidence indicates that such an approach can be beneficial [7].

In this study we aimed to arrive at insights more detailed than previously reported on effects of hypoxia on mitochondrial respiration in β-cells. For this purpose we used the INS-1 832/13 cell line and tested effects after an exposure to hypoxia that was followed by a re-oxygenation period. We first measured mitochondrial respiration in intact INS-1 832/13 cells. For detailed studies on respiration induced by electron flow through complex I and II we used permeabilized β-cells, permeabilization being necessary to allow access of mitochondrial substrates and to standardize levels of ADP in the cells. The respiratory findings were extended by functional and viability measurements and by measuring proteins of mitochondrial complexes in the INS-1 832/13 cells as well as in pancreatic islets from rats and humans.

## Methods and Design

### Materials

All materials were from Sigma-Aldrich, St Louis MO or from sources specified below.

### Cell line INS-1 832/13

Cells from the rat insulinoma cell line INS-1 832/13 were used in all respiratory experiments. This modification of the original INS-1 cell line [8] was developed and described in 2000 [9]. The cell line INS-1 832/13 is reportedly monoclonal and retains a strong insulin response to glucose over 66 population doublings, which accounts for ∼7.5 months of tissue culture. It can thus serve as an acceptable model for studying β-cell function under various physiological and pathophysiological conditions.

The INS-1 832/13 cells were grown as monolayer in 10 ml of RPMI-1640. The medium was supplemented with L-glutamine (4 mM), penicillin (100 IU/ml), streptomycin (100 μg/ml), HEPES (10 mM), sodium pyruvate (1 mM), mercaptoethanol (50 μM) and fetal calf serum (10%). The complete medium had a glucose concentration of 11 mM. The cells were cultured in a humidified incubator in an environment of 95% air and 5% CO_2_ at 37°C.

Cells were sub-cultured once a week through detachment with trypsin (0.01%) in ethylene-diaminetetraacetic acid (EDTA) (0.02%). The culture medium was changed every 3–4 days. For hypoxia experiments, cells were seeded into 25 cm2 flasks (Corning^QR^) in 4 ml medium and then cultured for three days before experiments.

Cell counting was performed before each respirometric experiment. Counting was done with a Countess automatic cell counter (Invitrogen, Carlsbad, California). A sample of the cell suspension (10 μl) was mixed (1:1) with trypan blue (0.4%, 10 μl) and the percentage of viable cells was calculated.

#### Hypoxic conditions

Hypoxia was achieved in a hypoxia chamber (Billups-Rothenberg Inc, Del Mar, CA) flushed with a gas mixture of 95% N2, and 5% CO_2_ together with a water-containing Petri dish. The O_2_ concentration in the hypoxia chamber was measured with an oxygen sensor, (Dräger Safety AG&Co., KGaA, Lübeck, Germany), placed inside the chamber. The duration of the hypoxia and re-oxygenation periods was based on our earlier hypoxia experiments on INS-1 832/13 cells (unpublished results). Cells were exposed to hypoxia (0.3–0.5% O_2_), usually for 18 hours, at 37°C and subsequently re-oxygenized for 20–22 hours at 37°C at normoxic conditions, defined as 95% air and 5% CO_2_. Control cells were kept in normoxic conditions during the whole experiment, i.e. for 38–40 hours.

#### Insulin secretion

Cells were seeded in 24-well plates (10^5^ cells/well). After three days of culture the medium was changed for all cells before cells were either challenged by hypoxia for 8 hours or unchallenged by culture under uninterrupted normoxic conditions. After re-oxygenation the medium was removed and cells were cultured for a further 2–3 hours in RPMI without glucose, supplemented with 20 mM HEPES and 1% FCS. Cells were then pre-incubated for 30 minutes in Krebs-Ringer bicarbonate buffer (KRB) with 10mM HEPES and 0.1% BSA in the absence of glucose. Final incubations were carried out in 0.5 ml of KRBH per well for 60–90 minutes with 3.3, 11 and 27 mM glucose (5 parallels per condition). Aliquots of media were secured for insulin RIA as described in [10]. Cellular insulin contents were measured after extraction of cell pellets with acid ethanol as described [11].

#### DNA quantification

DNA from samples of intact INS-1 832/13 cells was quantified by the Fluorescent DNA Quantification kit (Biorad, Hercules, CA).

#### ATP quantification

The ATP Bioluminescence Assay Kit CLS II (Roche Diagnostics GmbH, Mannheim, Germany) was used for the quantification of ATP in INS-1 832/13 cells. Measurements were performed during conditions identical to those preceding oximetry, i.e. in cells cultured in RPMI with 11 mM glucose.

#### Protein quantification

The Micro BSA^TM^ Protein Assay Kit (Thermo Scientific, Rockford, IL, USA) was used for the quantification of protein in lysates from INS-1 832/13 cells and human islets. Protein content in rat islets was confirmed by Bradford protein assay.

#### Western blots

Cells were trypsinized, washed two times in ice-cold phosphate-buffered saline (PBS). Cells (5 x 10^5^) were lysed on ice for 20–30 minutes in lysis buffer containing 150 mM NaCl, 50 mM Tris-HCl (pH 7.4), protease inhibitor cocktail (Complete Mini, Roche Diagnostics Gmbh, Mannheim, Germany), 1% Nonidet P40 (NP-40), 10% glycerol, 50 mM NaF and 1 mM Na_3_VO_4_. Protein extracts were denatured in loading buffer at room temperature for 20 minutes. Samples were analyzed on 12% SDS-PAGE gels run for 1 hour at 150 V before being transferred to nitrocellulose membranes for 1 hour at 250 mA. Membranes were blocked for 2 hours at room temperature with 5% (w/v) fat-free milk, 0.1% Tween 20 in Tris-buffered saline, pH 7.6, and then incubated over night at 4°C with primary antibodies for oxidative phosphorylation complexes MS604, (Mitosciences, USA) a “cocktail” of antibodies probing complexes 1–5; for complex 1 an antibody against CI subunit NDUFB8, and for complex 2 against SDHB at 1:500 dilutions and for beta-actin at 1:12,000 dilution. Monoclonal mouse anti-beta-actin was used as loading control. Secondary antibody incubations employed a HRP-linked anti-mouse antibody for 1 hour at room temperature. Immunoreactive bands were visualized using chemiluminescence (ECL Western blotting reagent, Pierce, Biotechnology, USA).

### High resolution respirometry (HRR)

Respirometric measurements were done by the Oxygraph-2k (OROBOROS, Innsbruck, Austria) instrument which makes use of Clark polarographic oxygen sensors. All calibrations and experiments were performed at 37°C with magnetic stirring set at 750 rpm. Chemicals for oximetry were used in the respirometric protocols as listed in Table 1. Chemicals were prepared, pH-adjusted and stored as recommended by OROBOROS INSTRUMENTS. Oxygen concentrations (nmol/mL) in the samples were measured and analysed by the Datlab software (OROBOROS INSTRUMENTS). Oxygen flux (pmol O_2_/s/10^6^ cells) was calculated as the negative time derivative of measured oxygen concentration.

**Table 1:** Reagents used in the different high-resolution respirometry protocols.

Chemicals	Role	Protocol
Malate	Substrate, CI	N/A
Pyruvate	Substrate, CI	N/A
Glutamate	Substrate, CI	SUIT_CI_, SUIT_CI+II_
Succinate	Substrate, CII	SUIT_CII_, SUIT_CI+II_
Cytochrome *c*	Substrate, CIV	SUIT_CI_, SUIT_CII_, SUIT_CI+II_
ADP+Mg^2+^	Substrate, CV	SUIT_CI_, SUIT_CII_, SUIT_CI+II_
FCCP	Protonophore, uncoupler	Protocol_Int_, SUIT_CI_, SUIT_CII_, SUIT_CI+II_
Rotenone	Inhibitor, CI	Protocol_Int_, SUIT_CI_, SUIT_CII_, SUIT_CI+II_
Antimycin A	Inhibitor, CIII	Protocol_Int_, SUIT_CI_, SUIT_CII_, SUIT_CI+II_
Oligomycin	Inhibitor, CV	Protocol_Int_
Digitonin	Permeabilization agent	SUIT_CI_, SUIT_CII_, SUIT_CI+II_

CI-CV: mitochondrial complexes I-V, ADP: adenosine diphosphate, FCCP: carbonyl cyanide-4-(trifluoromethoxy)phenylhydrazone, Protocol_Int_: protocol for intact cells, SUIT: substrate-uncoupler-inhibitor-titration protocol for electron flow through CI, CII and convergent CI+II.

For respirometric measurements, cell cultures in 25 cm^2^ flasks were first trypsinized and counted. For intact cells, a cell suspension was diluted with RPMI to an appropriate concentration of 10^6^ cells/ml. Then 2 ml samples of cell suspension were added to the Oxygraph chambers. For protocols in which cells were permeabilized, the cell suspension was re-centrifuged and the cell pellet re-suspended in the mitochondrial respiration medium MiR05 to the same concentration of 10^6^ cells/ml before being added to the Oxygraph chamber.

Each experiment was run with hypoxia-treated cells and controls in parallel. The use of a particular chamber for treated cells and controls was switched between experiments in order to avoid any bias caused by unresolved differences between chambers.

**Fig 1:**
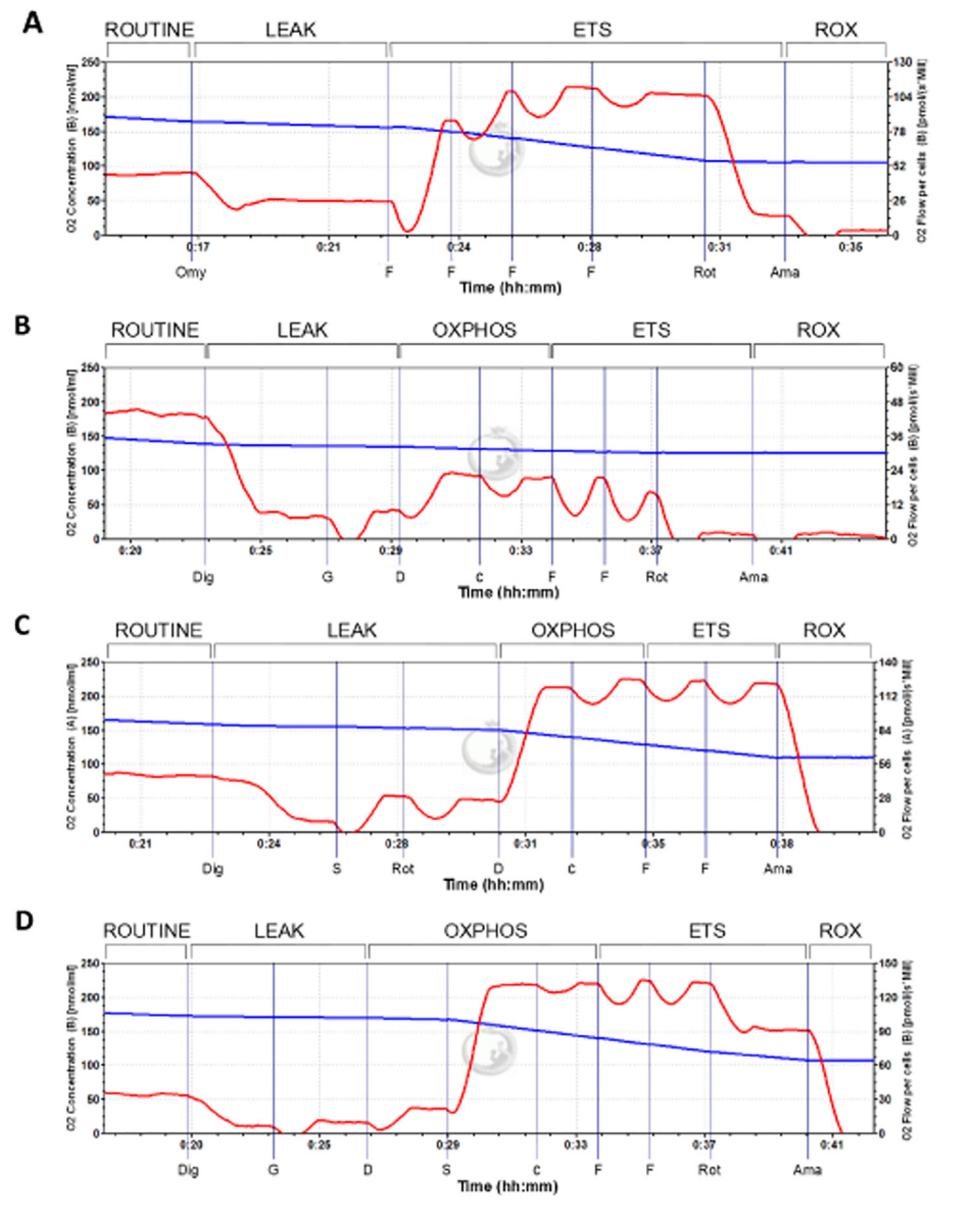
Oxygraphic example output of protocols with intact and permeabilized INS-1 832/13 cells (at normoxia). (A) Protocol with intact cells, (B) SUIT_CI_ protocol with permeabilized cells with glutamate as only reducing substrate, (C) SUIT_CII_ protocol with permeabilized cells with succinate as only reducing substrate, **(**D) SUIT_CI+II_ protocol with glutamate and succinate as reducing substrates. The blue line represents the oxygen concentration (nmol O_2_/mL) in the experimental chamber. The red line represents oxygen flux (pmol O_2_/s/10^6^ cells), the negative time derivate calculated from the measured oxygen concentration, normalized to the number of cells. ROUTINE: ROUTINE respiratory state of basal respiration, LEAK: LEAK respiratory state of uncoupled respiration, OXPHOS: OXPHOS respiratory state of maximum phosphorylative capacity, ETS: ETS respiratory state of the maximum capacity of the ETS, ROX: ROX respiratory state of residual oxygen consumption, Dig: digitonin, G: glutamate, D: ADP, S: succinate, c: cytochrome *c*, F: FCCP, Rot: rotenone, Ama: antimycin A.

Four different HRR protocols were used (Fig 1A–1D). One was designed for intact cells to provide information on the effect of hypoxia on basal and uncoupled respiratory rates, together with the enzymatic capacity of ETS. The three substrate-uncoupler-inhibitor titration (SUIT) protocols were designed for permeabilized cells to probe mitochondrial function by the addition of TCA cycle metabolites in saturating concentrations in different combinations, together with an uncoupler and ETS inhibitors. Using flux ratios derived from measured oxygen flux enabled internal normalization and relative contributions of substrate conditions, in particular coupling states, to be compared.

Exploratory experiments were performed on intact cells to rule out effects of such substrates that should enter cells only after permeabilization. We tested for possible effects on respiration in intact cells, (i.e. samples not treated by digitonin,) by malate (2 mM), glutamate (10 mM), succinate (10 mM), cytochrome C (4 mM) and ADP (5 mM). No stimulation of respiration by these substrates in intact cells was observed (results not shown).

#### Oximetry in intact cells

The respirometry protocol for intact cells, designated Protocol_Int_, was used as described earlier [12] for human islets. An example is given in Fig 1A. Basal respiration at the ROUTINE state was measured when a stable oxygen flux had been obtained following addition of cells into the experimental chamber. Uncoupled respiration in the LEAK state was measured when a steady oxygen flux had been attained following the addition of oligomycin (2 μM). Maximum capacity of the electron transfer system (ETS) was assessed by FCCP which was titrated in steps of 0.5 μM until a maximum oxygen flux was attained. To assess residual oxygen flux (ROX) due to non-respiratory side reactions, the inhibitors rotenone (0.5 μM) and antimycin A (2.5 μM), were added in sequence. Non-respiratory side reactions were corrected for by subtracting the oxygen flux measured in the ROX state. The protocol lasted 39 ± 9 minutes.

#### Permeabilization

Digitonin preferentially permeabilizes the cell membrane and only affects mitochondrial membranes at higher concentrations [13]. To permeabilize INS-1 832/13 cells, a cell suspension (2.2 ml) was first added to the Oxygraph chamber, followed by the subsequent addition of digitonin (8.1 μM, 10 μg/10^6^ cells). The optimal digitonin concentration for permeabilization of the cells was found in exploratory experiments (Results). Stable respiration in the presences of digitonin was awaited before continuing with the experimental protocol.

#### Mitochondrial respiration medium

The mitochondrial respiration medium MiR05 included EGTA (0.5 mM), MgCl_2_·6 H_2_O (3 mM), lactobionic acid (60 mM), taurine (20 mM), KH_2_PO_4_ (10 mM), HEPES (20 mM), D-Sucrose (110 mM) and BSA, essentially fatty acid free (1 g/l). The pH was adjusted to pH 7.1 as recommended by OROBOROS INSTRUMENTS.

#### Testing mitochondrial substrates in permeabilized cells

In exploratory experiments with glutamate (10 mM) and malate (2 mM) present, the addition of ADP (5 mM) did not stimulate respiration. The same was observed when using the lowest recommended concentration of malate (0.5 mM). Malate concentrations ranging from 125 μM to 0.5 mM, in the presence of ADP (2.5 mM) and in the presence and absence of glutamate failed to stimulate and, together with glutamate, seemed to depress respiration (S1 Fig). Based on these results, glutamate was chosen as the sole substrate providing electrons through complex I.

#### Substrate-uncoupler-inhibitor (SUIT) protocols

A SUIT protocol with converging substrate respiration was initially planned for this study. However, the absence of an effect by malate yielded a protocol with glutamate and succinate, a protocol which has been described for skeletal muscle [14]. Two other SUIT protocols were employed, using either glutamate or succinate as sole reducing substrate. Glutamate alone is not commonly used in respiratory protocols, while succinate alone is a common substrate condition in respirometric measurements [15].

#### Respiration through complex I

The protocol, designated SUIT_CI_, employs glutamate as the sole substrate. An example is given in Fig 1B. A stable ROUTINE state respiration was observed before adding digitonin (8.1 μM) to permeabilize the cells, attaining a State 1 respiration. The addition of glutamate (10 mM) yielded a State 2 LEAK respiratory state. An OXPHOS state was obtained by the subsequent addition of ADP (2.5 mM). Cytochrome *c* (10 μM) was added to assess mitochondrial membrane integrity. FCCP was titrated to assess ETS state respiration. Rotenone (0.5 μM) and antimycin A (2.5 μM) was added to investigate residual oxygen consumption in the ROX state. Rotenone abolished oxygen flux, confirming that the observed flux before administration of rotenone was only through complex I. Non-respiratory side reactions were corrected for by subtracting the oxygen flux measured in the ROX state. The protocol lasted 37.5 ± 7.5 minutes.

#### Respiration through complex II

This protocol, designated SUIT_CII_, utilizes succinate as the sole substrate. An example is given in Fig 1C. Here respiration was evaluated based only on electrons from complex II. First a ROUTINE and a state 1 eN respiration was established, then succinate (10 mM) and rotenone (0.5 μM) were added to attain a LEAK state in which any complex I-linked flux was inhibited by rotenone. ADP (2.5 mM) was added to attain an OXPHOS state, and cytochrome *c* (10 μM) was added to assess mitochondrial integrity. Then maximal non-coupled ETS respiration was obtained by titration of FCCP (0.5 μM steps). Residual oxygen flux of the ROX state was attained by addition of antimycin A (2.5 μM). ROX state flux was found to be negligible. The protocol lasted 37.5 ± 7.5 minutes.

#### Convergent respiration through complex I and II

This protocol, named SUIT_CI+II_, incorporates both glutamate and succinate as substrates for respiration. An example is given in Fig 1D. First a ROUTINE and subsequent state 1 eN respiration after titration of digitonin (8.1 μM) was obtained. Then, glutamate (10 mM) was added and a LEAK state was registered, before adding ADP (2.5 mM) yielding an OXPHOS state with electron flow from only complex I. Succinate was added to obtain an OXPHOS state with convergent electron flow from both complex I and complex II through the Q-junction, before cytochrome *c* was added to assess mitochondrial integrity. Subsequent titration of FCCP (0.5 μM steps) yielded maximal non-coupled ETS respiration with both glutamate and succinate as substrates. The subsequent addition of rotenone (0.5 μM) thus yielded the maximal non-coupled ETS respiration with complex I inhibited, i.e. with succinate as the only substrate. Antimycin A (2.5 μM) was added at the end of the protocol to evaluate the residual oxygen flux of the ROX state. ROX state flux was found to be negligible. The protocol lasted 48.5 ± 3.5 minutes.

#### Flux control ratios

Flux control ratios (FCRs) were calculated as coupling control ratios (CCRs) and substrate control ratios (SCRs) from the respirometry fluxes measured during the HRR protocols. Description of the CCRs is given in Table 2 and SCRs in Table 3.

**Table 2:** Definitions of the coupling control ratios (CCRs) used.

Ratio	Definition
LEAK CCR	LEAK respiration normalized to ETS capacity (L/E). It expresses the portion of maximum respiratory capacity that is due to proton leak. It increases from a theoretical minimum of 0.0, fully coupled, to 1.0, fully uncoupled.
ROUTINE CCR	ROUTINE respiration normalized to ETS capacity (R/E). It expresses how close the ROUTINE respiration operates to the maximum capacity of the system.
Net ROUTINE CCR	ROUTINE respiration without the LEAK component, normalized to ETS capacity ((R-L)/E).
Phosphorylation system CCR	OXPHOS respiration normalized to ETS capacity (P/E). It expresses how close maximum oxidative phosphorylation approaches the capacity of the ETS. A value of < 1.0 signify control by the phosphorylation system on the OXPHOS capacity. Consequently, OXPHOS capacity is not limiting if P/E = 1.0. P/E > 1.0 is regarded as an experimental artefact.
Phosphorylation CCR	LEAK respiration normalized to OXPHOS respiration (L/P). It expresses the efficiency of the phosphorylation system, i.e. the amount of flux in OXPHOS respiration that is due to proton leak.

L: LEAK state, E: ETS state, R: ROUTINE state, P: OXPHOS state.

**Table 3:** Definitions of the substrate control ratios (SCRs) used.

Ratio	Definition
Complex I Phosphorylation SCR	Complex I-linked OXPHOS respiration normalized to complex I + II-linked OXPHOS respiration (P_CI_/P_CI+II_). It expresses the portion of respiration in the OXPHOS state that is due to complex I-linked electron flow.
Complex II ETS SCR	Complex II-linked ETS capacity normalized to complex I + II-linked ETS capacity (E_CII_/E_CI+II_). It expresses the contribution of electron flow through complex II in the convergent ETS capacity state.

P_CI_: phosphorylative capacity with electrons through complex I, P_CI+II_: phosphorylative capacity with convergent electron flow from both complex I and II, E_CII_: ETS capacity with electrons from complex II, ETS_CI+II_: ETS capacity with convergent electron flow from both complex I and II.

### Rat and human islets

#### Rat islets

Male Sprague-Dawley rats were obtained from Scanbur (Sollentuna, Sweden). The rats had free access to water and a standard diet. At the time of experiments, the rats weighed 300–350 g. Rats were submitted to euthanasia by exposure to carbon dioxide. Pancreatic islets were isolated by collagenase digestion [16]. Islets were cultured free floating, at 37°C in a humidified atmosphere of 5% CO_2_ in air and RPMI medium containing 11 mM glucose and supplemented with 10% fetal calf serum, 2 mM L-glutamine, 100 IU/ml penicillin and 100 μg/ml streptomycin.

#### Human islets

Human islets were isolated using a modified semi-automated digestion method [17] from deceased donors at the islet isolation facility at the Section for Transplantation Surgery at Oslo University Hospital, Oslo, Norway, after appropriate consent was given for multi-organ donation. The islet viability was 80–95%. Islet preparations were those where quantitative insufficiencies had precluded their use for clinical transplantation. Islet preparations were cultured in CMRL 1066 medium (Mediatech Inc., USA) supplemented with 10% human AB serum (Milan Analytica, Rheinfelden, Switzerland), 2 mM L-glutamine, 100 IU/ml penicillin and 100 μg/ml streptomycin (all from Life Technologies). Upon arrival in Trondheim human islets were cultured before experiments similarly to rat islets, except for a lower glucose concentration (5.5 mM).

#### Hypoxia exposure in rat and human islets

Rat islets were exposed for 5.5 hours to hypoxia (rather than 18 hours as for INS-1 cells). Due to previous observations indicating increased susceptibility to damage by hypoxia [18], the oxygen concentration was kept at 2.8% rather than at < 1%. Human islets were also exposed to 5.5 hours of hypoxia. The degree of hypoxia in experiments with human islets was 0.8%. The period of re-oxygenation was 20–22 hours for rat and human islets alike (same as for INS-1 cells).

#### Viability measurements in rat and human islets

Apoptosis and necrosis: The viability of rat islets exposed to either hypoxia followed by re-oxygenation or to continuous normoxia (30 islets for each condition) was measured by quantification of necrotic and apoptotic DNA by the Cell Death Detection ELISA^PLUS^ kit (Roche Diagnostics, Gmbh, Mannheim, Germany).

MTT: The viability of human islets was assessed by the parameter of 3-(4,5-dimethyl-2-thiazolyl)-2,5-diphenyltetrazolium bromide (MTT). Islets exposed to either hypoxia followed by re-oxygenation or to continuous normoxia were handpicked (40 islets for each condition) into each of 2–5 wells on a 24 well plate for 4 hours of exposure to MTT. The MTT reagent was removed by washing the islets several times in 0.9% NaCl. Islets were then incubated for 1 hour in 400 μl DMSO per well at 37°C for color development. Fifty μl/well of 0.1 M NaCl in 0.1 M Glycine, pH 10.5, was added for color extraction. Two parallel aliquots per well were secured for absorbance (570 nm) measurements.

#### Insulin secretion, rat islets

After exposure to hypoxia followed by re-oxygenation or to continuous normoxia, islets were pre-incubated with 3.3 mM glucose in KRB for 30 minutes followed by batch incubations with 3.3 mM glucose or 16.7 mM glucose for 1 hour. Insulin accumulated in media was measured by RIA.

#### Western blots, rat and human islets

Western blotting was performed in cell lysates from rat islets as described [19]. Lysates from human islets were made by adding 1 μl of lysis buffer (as described above for INS-1 832/13 cells) per 5 islets. Both rat and human islets were handpicked and washed twice in ice-cold PBS, lysed and denatured in loading buffer at room temperature for 20 minutes. Samples were then analyzed by Western blot as described above for INS-1 832/13 cells.

### Statistics

The D’Agostino-Pearson omnibus K2 test was used to check all the raw data and their respective log-transformed values for Gaussian distribution. Data sets that were found to have a Gaussian distribution were tested for significance by the unpaired, two-tailed Student’s t test. Data sets that were not normal distributed, or had too few replicates to test for normality, were tested for significance by the non-parametric, two-tailed Mann-Whitney test, (except for tests done on OXPHOS state and state 1 fluxes in the exploratory experiments with glutamate, pyruvate and malate, where the Kruskal-Wallis and Dunn’s multiple comparison was used). For all other significance testing the Wilcoxon’s signed rank test or Student’s t-test was performed as appropriate. Results are presented as means ± SEM. A P-value < 0.05 was considered significant.

### Ethics

The protocols used with rat islets were approved by the Ethical Committee for Animal Research in Stockholm (Permit Number N88/11). The ethical guidelines of the Karolinska Institutet for the care and use of laboratory animals was followed. Human pancreata were obtained from brain-dead donors after verbal informed consent from relatives. Written consent is not sought, nor required according to the Health Authorities and Ethics Committees in Norway. The consent to donate was documented in the hospital record of the donor. The Regional Committee for Medical and Health Research Ethics Central in Norway approved the verbal consent procedure and the procedure of human islets and use of the tissue for research (2011/782).

## Results

### Effects of hypoxia on cell death, growth, insulin release and insulin contents

**Fig 2:**
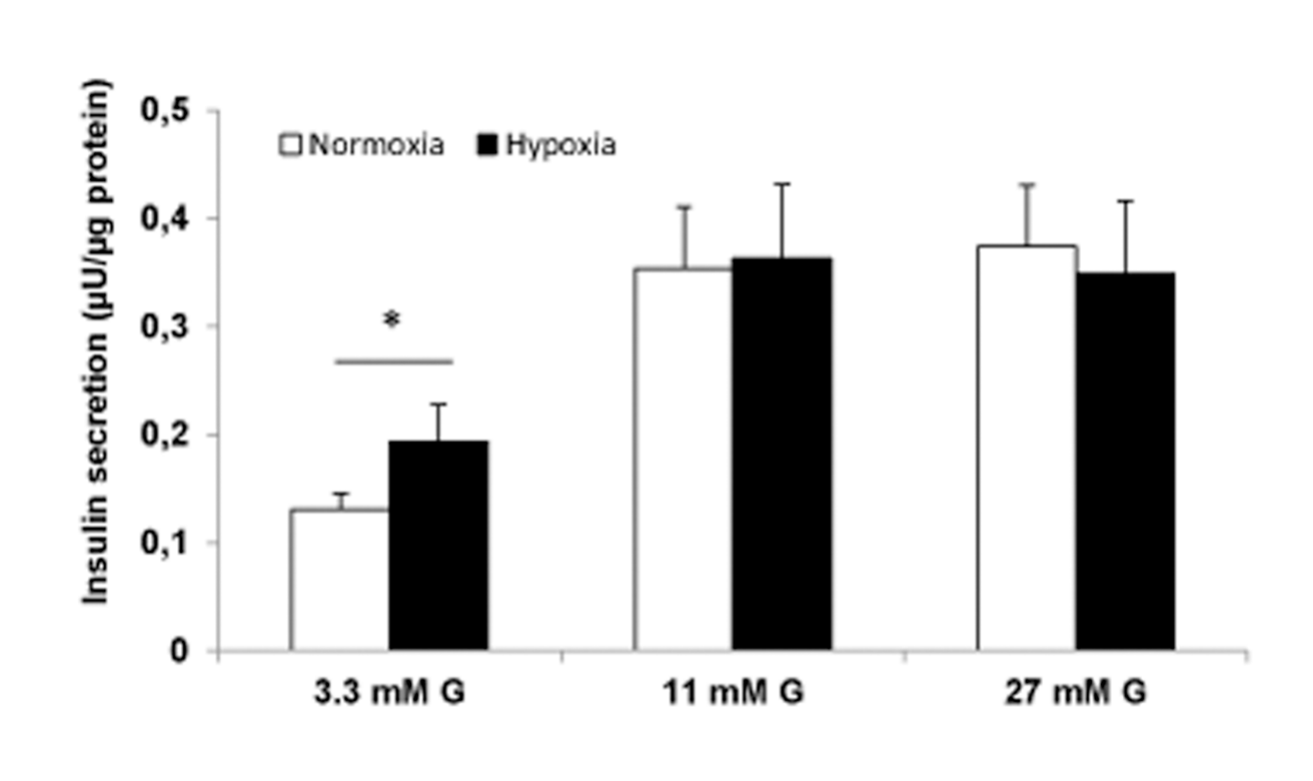
Previous hypoxia (8 hours) increased basal but not stimulated insulin secretion in INS-1 832/13 cells. Insulin release (final 60–90 min incubations) with 3.3, 11 and 27 mM glucose (G). Protein content was estimated from a mean of three measurements in each experiment. Data are mean ± SEM, n = 5, *P < 0.05.

Exposure to hypoxia for 18 hours reduced numbers of viable INS-1 832/13 cells to 1.74 ± 0.15 million cells, compared to controls (6.11 ± 0.37 million cells). After 20 hours of re-oxygenation, the cell number increased to 3.32 ± 0.23 and 8.12 ± 0.26 million cells for hypoxia-treated (n = 17) and controls (n = 13), respectively. This increase was more marked in hypoxia treated cells (45 ± 7%) than in control cells (21 ± 4%). All cell suspensions used in the respiratory experiments showed a viability of > 97%, given by the trypan blue parameter. Exposure to hypoxia for only 8 hours led to lesser damage with a 25.8 ± 4.8% loss of viability when measured after re-oxygenation (P < 0.02, n = 7). Parallel measurements of insulin secretion (basal and stimulated) in five separate experiments showed an increase in basal secretion in hypoxia (8 hours) exposed cells: 0.194 ± 0.034 μU/μg protein for hypoxia vs. 0.131 ± 0.015 μU/μg protein for normoxia, P < 0.05. However, glucose-induced insulin secretion post hypoxia was not reduced. Results are shown in Fig 2.

In additional experiments (n = 3) we related secretion to cellular insulin contents. Compatible with the data for insulin secretion related to protein content, we found a tendency for increased basal insulin secretion post hypoxia (4.6 ± 0.9% for hypoxia vs. 2.9 ± 0.5% for normoxia, P < 0.14) and preserved glucose-stimulated insulin secretion (12.4 ± 2.1% for hypoxia vs. 11.8 ± 2.5% for normoxia).

### ATP contents

**Fig 3:**
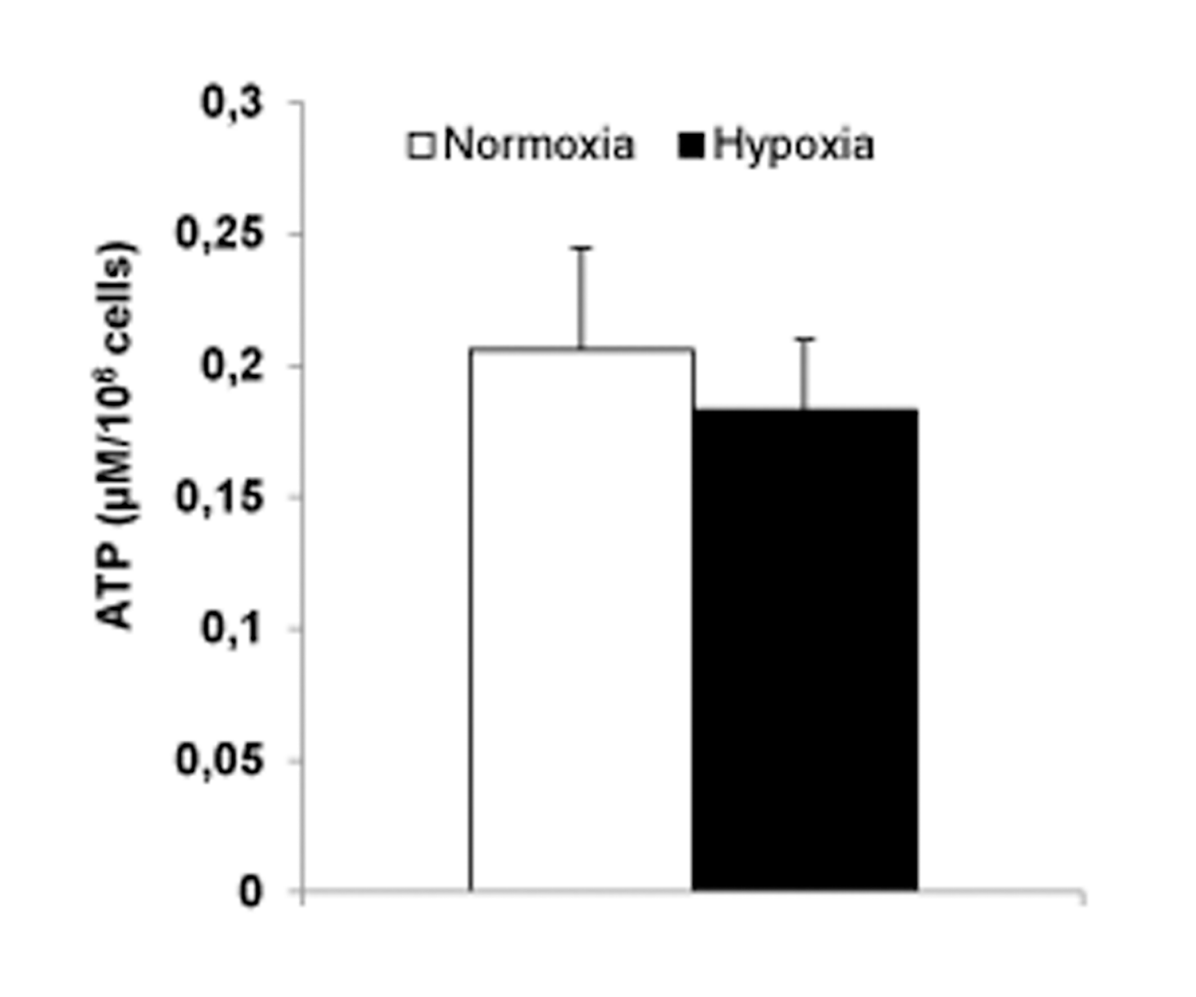
Previous hypoxia (8 hours) failed to affect levels of ATP in INS-1 832/13 cells. Cells were cultured in RPMI with 11 mM glucose. Data are mean ± SEM based on five separate experiments (three parallels per experimental condition).

Measurements were performed in cells cultured in RPMI and 11 mM glucose. Previous hypoxia (8 hours) failed to t affect levels of ATP (Fig 3).

### Effects of hypoxia on respiration in intact INS-1832/13 cells

#### After 18 hours of hypoxia

**Fig 4:**
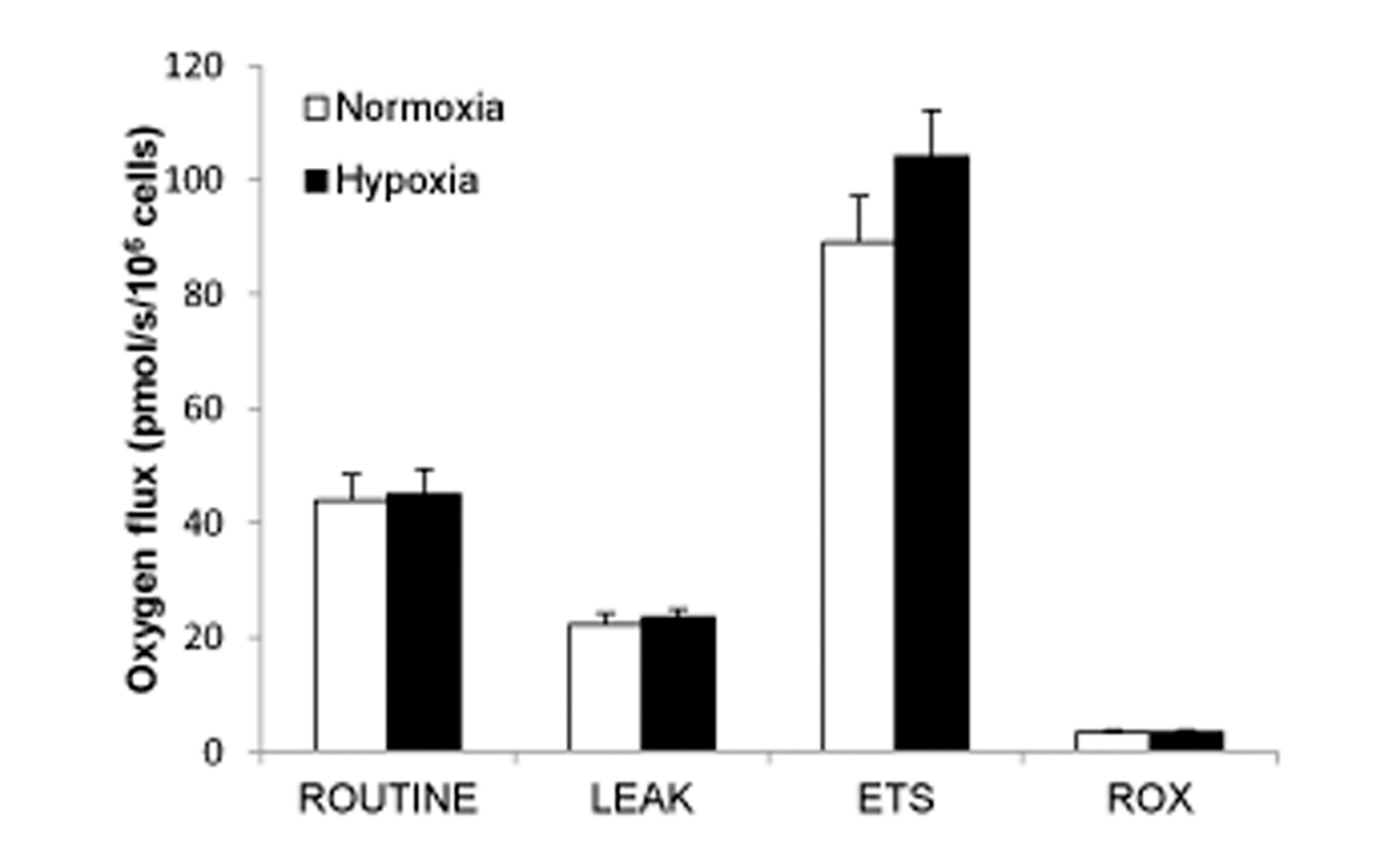
Oxygen flux in different respiratory states in intact INS-1 832/13 cells. Sequential respiratory states in the Protocol_Int_ protocol, comparing hypoxia with normoxia ROUTINE: basal respiration measured at physiological substrate conditions, before any reagent additions, LEAK: uncoupled respiration after addition of oligomycin, ETS: flux capacity of the electron transfer system, induced by FCCP, ROX: residual oxygen consumption, a measure of oxidative side reactions obtained by adding rotenone and antimycin A to inhibit complex I and II, respectively. Fluxes are expressed as means ± SEM of five experiments (comprising totally 12 single measurements),*P < 0.03.

The fully uncoupled respiration, i.e. ETS state respiration, in intact cells was significantly higher in hypoxia-exposed cells; 108.3 ± 4.4 pmol O_2_/s/10^6^ cells for hypoxia and 94.2 ± 4.6 for normoxia (P < 0.03, n = 5) (Fig 4). ROUTINE and LEAK state respiration was not significantly affected by the hypoxia treatment.

Hypoxia-treated cells displayed a significant lower ROUTINE control ratio, indicating that a smaller fraction of maximum ETS capacity is used for basal respiration after hypoxia (Table 4). This is also indicated by a decreased net ROUTINE control ratio (P < 0.05). A reduction of the LEAK control ratio, which indicates less uncoupling, was also significant (P < 0.05). However, the L/R ratio was not significantly affected.

**Table 4:** Coupling control ratios (CCRs) derived from the protocol with intact cells.

Coupling control ratio	Normoxia	Hypoxia	Definition
LEAK/ROUTINE ratio	0.517 ± 0.040	0.537 ± 0.040	L/R
ROUTINE control ratio	0.493 ± 0.014	0.435 ± 0.024*	R/E
LEAK control ratio	0.250 ± 0.022	0.230 ± 0.014*	L/E
Net ROUTINE control ratio	0.239 ± 0.020	0.205 ± 0.024*	(R-L)/E

L: LEAK state, E: ETS capacity, R: ROUTINE state. Ratios are means ± SEM of five experiments (comprising totally 12 single measurements)

*P < 0.05.

#### After 8 hours of hypoxia

In a separate series of experiments we employed 8 hours of hypoxia followed by re-oxygenation. As indicated above (Results first paragraph) viability measured from surviving cells was much less compromised (26% decrease) than after 18 hours of hypoxia (72% decrease). Despite the lesser impact of hypoxia after 8 hours of exposure the oxymetric results were similar to those seen after 18 hours of hypoxia. Hence, respiratory capacity was increased; 64.6 ± 5.5 at normoxia vs. 73.8 ± 3.7 pmol/s/10^6^ cells at hypoxia, (P < 0.02, n = 6). Similar results were obtained when expressed per DNA contents (see S1 Text). Also the LEAK control ratio was significantly reduced after previous hypoxia, (0.29 ± 0.021 at normoxia vs. 0.26 ± 0.012 after hypoxia, (P < 0.05, n = 5) indicating, again, lesser uncoupling.

### Permeabilized cells: Effects of experimental medium on basal respiration

The MiR05 medium was used in cells to be permeabilized. However, oxygen flux was first allowed to stabilize in the ROUTINE states while the cells were still intact. This permitted a comparison of basal respiration using RPMI and MiR05 media. The type of medium had significant effects on basal respiration for control cells (47.9 ± 2.4 and 33.0 ± 1.0 pmol O_2_/s/10^6^ cells for RPMI and MiR05, respectively) and for hypoxia-exposed cells (48.3 ± 1.9 and 40.2 ± 0.9 pmol O_2_/s/10^6^ cells for RPMI and MiR05, respectively). Interestingly, basal respiration in intact cells in the MiR05 medium was thus significantly (P < 0.001, n = 12–13) increased by hypoxia in the MiR05 but not in the RPMI medium (n = 11).

### Permeabilized cells: Optimal concentration of digitonin

The optimal concentration of digitonin was tested for in two separate experiments (S2 Fig). Titration of digitonin was done in the presence of, succinate (10 mM), ADP (5 mM) and rotenone (0.5 μM) was added. The concentration yielding the highest oxygen flux through mitochondrial complex II was chosen. The optimal concentration of digitonin and found to be 8.1 μM (10 μg/10^6^ cells) in 2 mL MiR05 medium. The addition of cytochrome *c* to permeabilized cells in the subsequent SUIT-protocols did not significantly increase O_2_ flux (data not shown).

### Permeabilized cells: Lack of respiratory response to malate

When glutamate and malate were present, then the further addition of ADP had no stimulatory effect on respiration. The same was observed when using the lowest recommended concentration (0.5 mM) of malate. Since malate did not have a stimulatory effect on respiration at both ends of the recommended concentration spectrum (0.5 to 2 mM), titration protocols were performed to evaluate if a lower malate concentration could still be stimulatory. A titration protocol with malate concentration ranging from 125 μM to 500 μM, in the presence of ADP, and both in the presence and absence of glutamate, was carried out. Malate alone caused no stimulation of respiration, and together with glutamate seemed to depress respiration S1 Fig).

**Fig 5:**
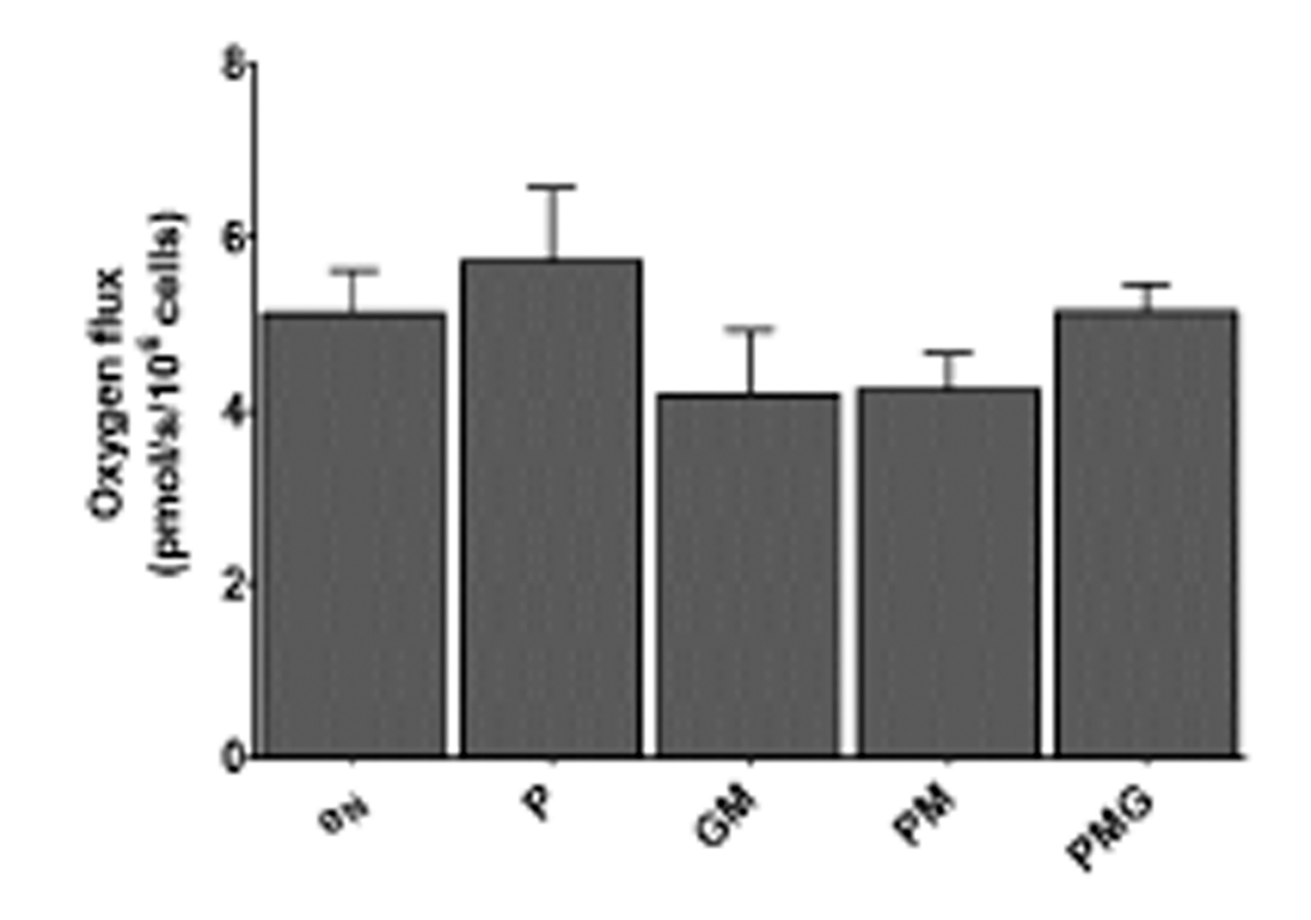
Phosphorylative capacities of different NADH-linked substrate combinations. The graph shows the ability of different substrate combinations of pyruvate (5 mM), glutamate (10 mM) and malate (0.5 mM) to stimulate respiration in the presence of ADP (2.5 mM). State 1 (e_N_) is used as a control state. No difference was found between state 1 respiration and the OXPHOS states of the different substrate conditions. e_N_: endogenous respiration without reducing substrates or ADP (six measurements), P: pyruvate (three measurements), GM: glutamate and malate (six measurements), PM: pyruvate and malate (three measurements), PMG: pyruvate, malate and glutamate (three measurements). Fluxes are expressed as means ± SEM.

These observations led to titration experiments with the administration also of pyruvate in different combinations with malate and glutamate to investigate if any of these substrate conditions would support respiration in the presence of ADP. No difference (P *>* 0.05) was found between state 1 respiration and the OXPHOS states of the different substrate conditions (Fig 5). Oxygen flux with malate together with pyruvate and glutamate did not stabilize, prohibiting measurement of a steady-state. These observations led to the use of glutamate as the sole substrate for providing electrons to complex I.

### Permeabilized cells: Respiration through complex I

The LEAK and OXPHOS state flux and ETS capacity was compared in experiments with hypoxia-exposed cells and control cells with the NADH-linked substrate glutamate as only source of electrons, thus ensuring electron flow only through complex I. An example of this protocol is given in Fig 1B. The phosphorylation capacity of complex I-linked electron flow was significantly elevated by the previous exposure to hypoxia treatment (13.3 ± 1.3 and 21.0 ± 1.3 pmol O_2_/s/10^6^ cells in normoxia and hypoxia, respectively; P < 0.05, n = 4). Maximum capacity of the ETS was increased as indicated by the significant increase of flux in the ETS state (14.5 ± 1.3 and 20.4 ± 1.0 pmol O_2_/s/10^6^ cells in normoxia and hypoxia, respectively, P < 0.05, n = 4). There was no change in oxygen flux due to oxidative side reactions, given by ROX respiration.

### Permeabilized cells: Respiration through complex II

LEAK and OXPHOS state flux, and ETS capacity, was compared in hypoxia-exposed cells and control cells, with the FADH_2_-linked substrate succinate as the only source of electrons, thus ensuring electron flow only through complex II (example in Fig 1C). ROX state flux was found to be negligible.

Hypoxia significantly increased the capacity of ETS (107.9 ± 6.1 pmol O_2_/s/10^6^ cells for hypoxia and 89.1 ± 3.0 and for normoxia P < 0.04, n = 4 or 5). Complex II-linked phosphorylation capacity showed a moderate increase (101.9 ± 6.7 pmol O_2_/s/10^6^ cells for hypoxia and 87.6 ± 3.7 and for normoxia, though not a significant one (P = 0.19, n = 4 or 5).

### Permeabilized cells: Convergent respiration through complexes I and II

The third SUIT protocol, SUIT_CI+II_, evaluated respiration with electron flow through both complex I and II in hypoxia-exposed cells and control cells, with converging electron flow to ubiquinone. An example is given in Fig 1D.

With glutamate and succinate as substrates, respiration differed only slightly and non-significantly in normoxia vs. hypoxia-exposed cells in the convergent phosphorylation state (117.7 ± 7.5 and 124.5 ± 3.8 pmol O_2_/s/10^6^ cells in normoxia and hypoxia, respectively; P = 0.69, n = 4), in the uncoupled state with convergent electron flow through mitochondrial complex I and II (121.6 ± 7.7 and 134.2 ± 3.2 pmol O_2_/s/10^6^ cells in normoxia and hypoxia, respectively; P = 0.34, n = 4) and in the uncoupled state with inhibition of complex I (83.3 ± 4.2 and 87.0 ± 1.9 pmol O_2_/s/10^6^ cells in normoxia and hypoxia, respectively; P = 0.69, n = 4).

### Permeabilized cells: Flux control ratios

Coupling and substrate control ratios derived from the three SUIT protocols (Table 5) indicated that the phosphorylation system exerts relatively little control over the phosphorylation capacity in control cells (normoxia throughout), the P/E-ratio being > 0.9. Hypoxic treatment led to a significant decrease in phosphorylation capacity control with electron flow only through complex I. Such seemed not the case for electron flow through complex II and convergent flow through both complex I and II, as the ratio tended to decrease (P = 0.11 and P = 0.20 for complex II and both complexes, respectively).

**Table 5:** Coupling and substrate control ratios derived from the SUIT protocols.

Control ratio	Normoxia	Hypoxia
**Coupling control ratios**		
*Complex I*		
L_CI_/E_CI_	0.418 ± 0.024	0.364 ± 0.030
L_CI_/P_CI_	0.448 ± 0.017	0.381 ± 0.018*
P_CI_/E_CI_	0.918 ± 0.012	1.025 ± 0.024*
*Complex II*		
L_CII_/E_CII_	0.264 ± 0.007	0.225 ± 0.005*
L_CII_/P_CII_	0.265 ± 0.006	0.238 ± 0.006*
P_CII_/E_CII_	0.982 ± 0.003	0.944 ± 0.019
*Complex I+II*		
P_CI+II_/E_CI+II_	0.968 ± 0.008	0.928 ± 0.019
**Substrate control ratios**		
P_CI_/P_CI+II_	0.160 ± 0.009	0.147 ± 0.002
E_CII_/E_CI+II_	0.687 ± 0.014	0.648 ± 0.006*

CI: complex I, CII: complex II, L/E: LEAK control ratio, L/P: phosphorylation control ratio, P/E: phosphorylation system control ratio, P_CI_/P_CI+II_: substrate control ratio relating phosphorylative capacity through complex I with phosphorylative capacity by convergent electron flow, E_CII_/E_CI+II_: substrate control ratio relating maximum ETS capacity through complex II with capacity by convergent electron flow. Ratio are expressed as means ± SEM, based on four-eight measurements

*P < 0.05.

The LEAK control ratio (L/E) was reduced in cells exposed to hypoxia, indicating less uncoupling. Also less uncoupling is suggested by the significantly decreased phosphorylation control ratio (L/P) in both complex I and complex II-linked respiration.

The substrate control ratio relating non-coupled respiration through complex II with the convergent non-coupled respiration (E_CII_/E_CI+II_) showed a significant decrease in hypoxia- treated cells, indicating a decreased electron transfer capacity through complex II compared to the convergent electron flow through the Q-junction.

### Effects on proteins of mitochondrial complexes

**Fig 6:**
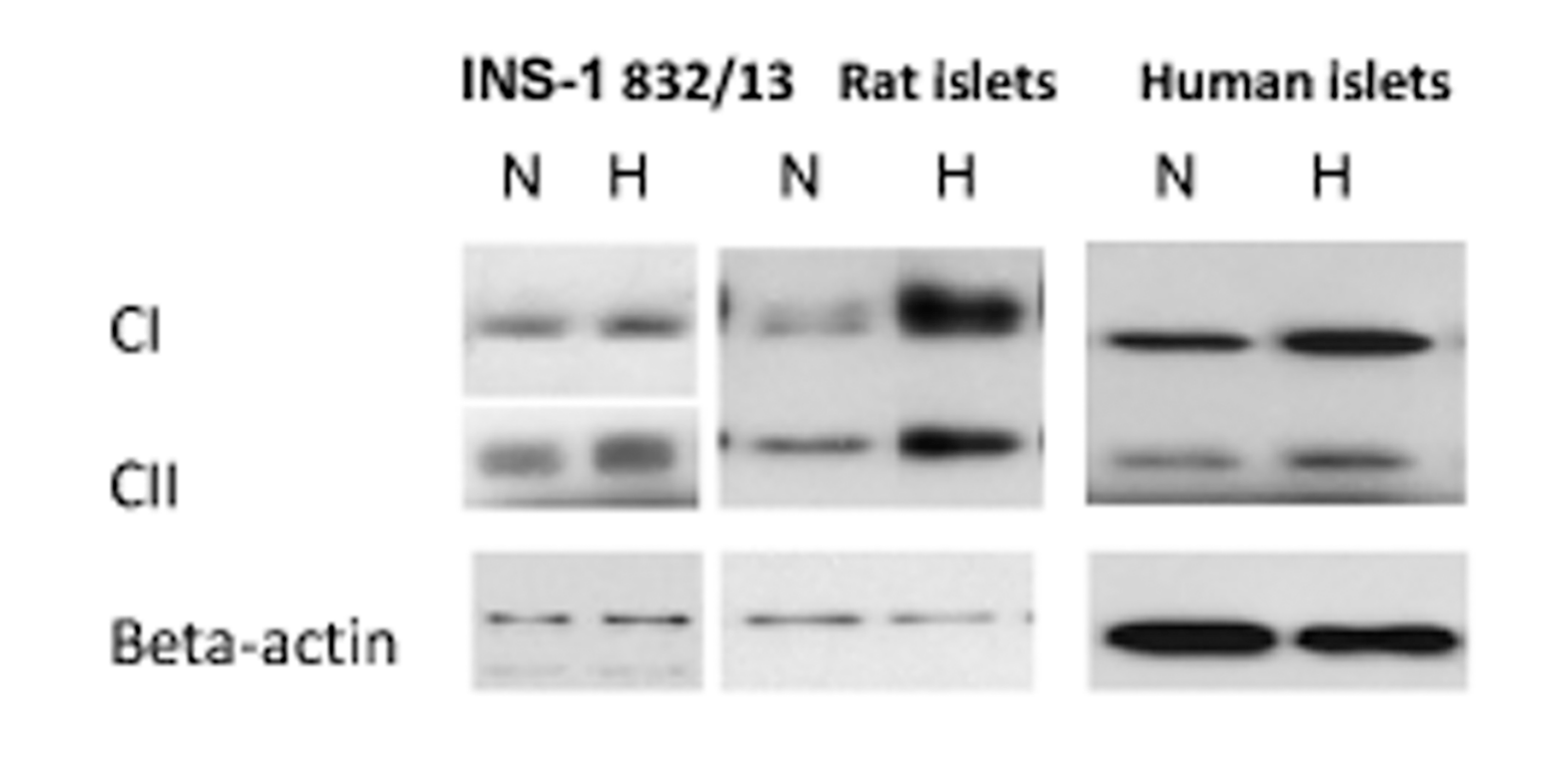
Effects of hypoxia on mitochondrial complexes I and II. Immunoblotting of subunits of the complexes were performed on cells and islets that were harvested after hypoxia followed by re-oxygenation (H) or continuous normoxia (N). Representative Western blots are shown.

Because oximetry overall indicated that hypoxia increased respiratory capacity we sought to investigate whether mitochondrial complexes were correspondingly up-regulated. As shown in Fig 6 and Table 6 such was indeed the case for proteins of complexes I and II in hypoxia-treated INS-1 cells. To clarify whether up-regulation by hypoxia was also seen in native cells we tested for such effects in rat and in human islets. Up-regulation by previous hypoxia of proteins of complexes I and II was then found both in rat and in human islets (Fig 6 and Table 6).

**Table 6:** Effect of hypoxia on mitochondrial complex protein levels.

Amount of protein complex subunits I and II after hypoxia exposure(as % of protein levels at normoxia)			
	(n)	CI-NDUFB8	CII-SDHB
INS-1 832/13	4	130 ± 9*	136 ± 10*
Rat islets	8	134 ± 17	120 ± 9*
Human islets	4	151 ± 13*	176 ± 13*

Complex (C) I and II subunits; NDUFB8: NADH dehydrogenase [ubiquinone] 1 beta subcomplex subunit 8, SDHB: succinate dehydrogenase subunit B. Islets from eight rats and two human donors were used for the experiments. Data are means ± SEM

*P < 0.05 for the effect of hypoxia.

### Functionality of islets processed for Western blotting

To assess the functionality of cells processed for Western blotting we measured in parallel experiments glucose-induced insulin secretion. As partly reported [14] for rat islets exposed for 5.5 hours for 2.8% oxygen followed by re-oxygenation insulin release was increased during basal conditions (1.7 ± 0.3 μU/islet/h following hypoxia vs. 0.8 ± 0.1 μU/islet/h following normoxia (P < 0.003, n = 8) whereas no negative effects were observed for glucose-induced insulin secretion (91.6 ± 6.7 μU/islet/h following hypoxia vs. 90.5 ± 6.4 μU/islet/h for continuous normoxia, n = 8). For human islets similar findings (significant increase in basal secretion, no effect of glucose-induced insulin secretion) were previously reported after 8 hours of exposure to hypoxia [12]. For effects on viability in rat islets we measured apoptotic and necrotic DNA. Islets exposed to 5.5 hours of 2.8% oxygen followed by re-oxygenation displayed a 1.55 ± 0.16 fold increase (p < 0.02) in of necrosis and a 4.30 ± 0.74 fold increase (P < 0.003) of apoptosis, n = 8.

Viability of human islets was assessed by MTT. A 5.5 hour period of 0.8% oxygen followed by re-oxygenation reduced the MTT signal by 12.3 ± 3.8% (ns, n = 4).

## Discussion

### Overall effects

This study focused on lingering effects of hypoxia in vitro on mitochondrial function in clonal insulin-producing cells, both intact and permeabilized, of the cell line INS-1 832/13. These results provide evidence for hypoxic adaptation in the form of increased capacity for mitochondrial ATP production and electron transfer, together with increased coupling efficiency. Compatible herewith are increases in subunits of complexes 1 and 2, a finding that extends to islets of rat as well as human islets.

### Viability

The extent of exposure to hypoxia in INS-1 832/13 cells (usually 18 hours) was clearly detrimental since there was considerable loss of cells. After re-introducing the surviving cells to a normoxic environment, cell growth resumed at a seemingly higher rate than in control cells. This might be attributed to a lower confluence that supports higher growth rate as a result of less inhibitory cell-cell communication.

The surviving cells could represent a selection of cells that managed to initiate sufficient adaptive changes in mitochondrial function. However, very similar adaptive responses (discussed below) were observed in cells that had been exposed to hypoxia for only 8 hours followed by much reduced damage. Also the notion of selection is not favored by the fact that the cells used are reportedly monoclonal [9].

### Results in intact cells

Uncoupled respiration constitutes a large part of the basal respiratory rate in rodent islets and β-cells [20, 21]. In agreement herewith we find a high degree of uncoupling in intact INS-1 832/13 cells. Hence, uncoupled respiration accounted for 46.7–52.5% of basal respiration.

The respiratory effects of hypoxia and re-oxygenation on intact INS-1 832/13 cells were modest but significant. Hypoxia led to a slight elevation of maximum ETS capacity, with subsequent reduction of the ROUTINE control ratio. The ROUTINE control ratio is a measure of basal respiration normalized to maximum capacity. Decreased ATP demand and ADP-stimulated basal respiration is one possible cause for a reduction, decreased uncoupling another, limitation of oxidative capacity by defects of substrate oxidation and complexes of the ETS a third [15]. The finding of a decreased LEAK control ratio supports reduced proton leak as a possible factor. Decreased uncoupling in intact cells is also in line with our findings in permeabilized cells, discussed below.

The increase in maximum ETS capacity in intact INS-1832/13 cells indicates adaptation to hypoxia by increasing levels and activities of mitochondrial complexes. These observations differ from the global depression of respiration in intact rat islets, previously reported [18]. However, adaptation (up-regulation of mitochondrial complexes) could be demonstrated in rat islets by employing here a lesser degree of hypoxia than in previous experiments (Fig 6) in a setting in which glucose-induced insulin secretion was not affected. In this context, it should be noted that the degree of uncoupling could not be evaluated in rat islets, since the effect of oligomycin on oxygen flux did not stabilize [18].

We did not observe any decrease in ATP levels due to previous hypoxia. This finding agrees with previous measurements (expressed per DNA) in rat islets after hypoxia followed by re-oxygenation [18]. Increased efficiency of mitochondrial oxidation could be expected to raise ATP levels. However, increased expenditure of ATP could result in unaltered levels of ATP. Insulin secretion and biosynthesis are energy requiring processes, as are also the electrolyte pumping mechanisms which serve to uphold intra to extracellular differences in potassium and calcium levels. Further studies are warranted to explore possible hypoxia-induced effects on the energy expenditure by the mentioned factors.

### Effects of media

In intact cells basal respiration was significantly depressed in the MiR05 medium compared to RPMI medium. This was to be expected as glucose is absent in MiR05 and Ca^2+^ availability is controlled by the chelator EGTA. More importantly a significant elevation of basal respiration was seen in cells exposed to hypoxia compared to controls in the MiR05 but not in the RPMI medium. This difference could suggest an increased efficiency in utilizing endogenous substrates after hypoxia; however, biochemical and molecular mechanisms remain to be elucidated.

### Substrate effects in permeabilized cells: Malate not usable

The combination of malate and glutamate is a well-established method to stimulate complex I-linked respiration in many different tissues and cells [15]. However, we observed only inhibition by malate, even at low concentrations. Results [22] of a recent study in INS-1E cells, which is another subtype of the INS-1 cell line, appear compatible with our results. The previous study found that glutamate and malate together could not support respiration in INS-1E cells, nor could pyruvate, isocitrate and α-ketoglutarate together with malate. A possible biochemical explanation of this lack of respiratory stimulation, or frank inhibition, by malate is the high activity of the malate-aspartate shuttle in β-cells, as NADH re-oxidation to NAD^+^ is paramount for maintaining a high glycolytic activity [23]. Increases in malate concentration outside the mitochondrion may facilitate increased transport of α-ketoglutarate out of the matrix through anti-port with malate. Increases in matrix malate will equilibrate with fumarate formation, depressing oxidation of succinate to fumarate, and cause subsequent increase of oxaloacetate formation, oxaloacetate being a potent inhibitor of succinate dehydrogenase [24]. However, more research is needed to ascertain the bioenergetic processes underlying this distinct characteristic of the β-cells studied here.

### Effects of glutamate and succinate

Glutamate alone could support respiration, but to a much lower degree than succinate alone. This agrees with succinate being a potent inducer of phosphorylation also in β-cells [22, 25]. An analogue of succinate, methyl succinate, which is permeable to membranes, stimulates insulin secretion in rat islets and INS-1 cells in a manner similar to that of glucose, emphasizing the importance of succinate in the context of β-cell metabolism as a signal for secretion [26].

The coupling control ratios calculated from the respiratory data indicate that β-cell mitochondria respire at a higher efficiency with succinate than with glutamate. This finding is in line with previous observations in other types of cells of showing higher leak and ROS production by complex I activity [27].

### Effects of hypoxia in permeabilized cells

Respiration was elevated in the phosphorylation and uncoupled steady states of cells exposed to hypoxia, compared to control cells. This was most apparent in NADH-linked respiration with glutamate as substrate. On balance, our data indicate a moderately increased absolute respiration. The previous exposure to hypoxia also seemed to increase coupling efficiency, indicated by the decreased LEAK control ratio in complex II-linked respiration (L_CII_/P_CII_), and decreased phosphorylation control ratio in both CI (L_CI_/P_CI_) and CII-linked (L_CII_/P_CII_) respiration. Our findings are in line with previous findings [28] in rat liver and in brine shrimp embryo, however those measurements were done during acute hypoxia and not, as here, after a hypoxic period with subsequent re-oxygenation. In general, adaptation to severely reduced access to oxygen during hypoxia should be beneficial by reducing as much as possible futile oxygen consumption as a result of proton leak.

The phosphorylation system CCR seems to have little control over the phosphorylation capacity in the INS-1832/13 cells, i.e. the enzymatic capacity of utilizing the proton-motive force is near to the capacity of electron transfer. Hypoxia followed by re-oxygenation seems to totally abolish the control over complex I-linked oxidative capacity. This is indicated by a phosphorylation system control ratio (P_CI_/E_CI_) ∼1.0. However, complex II-linked oxidative capacity when analyzed separately, displayed slightly depressed capacity of substrate oxidation compared to ETS capacity (P_CII_/E_CII_) after hypoxia. This might be an adaptation to a shift in metabolism to NADH oxidation as HIF activation during hypoxia causes an up-regulation of glycolytic activity, resulting in more NADH being produced from NAD^+^ during glycolysis [2]. Without subsequent up-regulation downstream to accommodate for increased NADH production upstream of ETS, this could potentially become a limiting factor.

The SCR relating complex I-linked phosphorylation with converging complex I- and II-linked phosphorylation (P_CI_/P_CI+II_) was not significantly affected by the hypoxic treatment. Thus, a shift in complex I-linked phosphorylation capacity compared with converging capacity was not observed, at least not with glutamate as reducing substrate.

A significant reduction was seen with the SCR in hypoxia-exposed cells relating ETS capacity for complex II-linked substrates with converging ETS capacity (E_CII_/E_CI+II_). This is an indication of reduced enzymatic capacity for complex II-linked electron flow compared to converging flow.

### Proteins of mitochondrial complexes

The enhanced respiratory capacity that was induced by hypoxia prompted measurements of proteins of mitochondrial complexes. The increases in proteins (subunits) of mitochondrial complexes seen after hypoxia in INS-1 cells are in line with, and extend, the findings on respirometry. Further, we demonstrate similar effects in rat and in human islets, something which implies that also the respirometric findings may, in part at least, and under certain conditions be extrapolated to native islet cells. In this context it should mentioned that technical problems using islets preclude the detailed respirometric measurements that were performed in the INS-1 cells.

### Effects on functionality

How does a measured exposure to hypoxia affect the secretion of insulin,—unquestionably the major function of beta cells? In line with the notion of adaptation we find no negative effect on the stimulatory response to glucose (measured in absolute terms), however basal secretion at low glucose is increased Quite similar effects of previous hypoxia (unaltered stimulated, increased basal secretion) are observed in rat and human islets. Increased basal secretion after hypoxia may thus be a universal finding. It will be of interest in future studies to dissect molecular mechanisms behind increased basal secretion and possible linkage to the changes in mitochondrial function that we demonstrate here.

### Limitations

Certain limitations should be recognized. All hypoxia experiments in this study were done after 20–22 hours of re-oxygenation. This was to evaluate respiratory adaptive responses in cells that survived a severe hypoxic exposure for an extended period of time. By only measuring respiration after re-oxygenation we fail to detect such responses that might dissipate during re-oxygenation. However, measurements performed immediately after a hypoxic exposure will be obtunded by cells undergoing necrosis or apoptosis. In pilot experiments performed immediately after 18 hours of hypoxia we observed considerable release of cytochrome *c* (results not shown). Cytochrome *c* is released as an intermediate in apoptotic signaling, causing ambiguity when determining the effect of the permeabilization and cell treatment on mitochondrial integrity

The present study normalized oxygen flux to the number of cells, not taking into account a potential change in mitochondrial density and upstream metabolic changes. More information on the absolute respiratory rates would be provided by normalizing respiratory data to amount of mitochondrial DNA, activity of citrate synthase or production of CO_2_. This, on the other hand, would not affect relative flux ratios.

β-cells co-exist with four other endocrine cell types in pancreatic islets. A population of pure β-cells thus differs from the in vivo situation. This can be viewed as a limitation; on the other hand, there are benefits of using β-cells in respirometric measurements, as opposed to islets. Hence, if the aim is to selectively measure β-cell function, then non β-cells present in islets of Langerhans will confer ambiguity. This is particularly relevant with regard to hypoxia, since α-cells are reportedly more resistant to damage by hypoxia [29]. Using cells rather than islets also eliminates the possibility of an oxygen gradient forming, and provides easy-to-read flux plateaus as respiratory states. Another aspect to consider is the difference in oxygenation of cell cultures and cultured islets. Cultured INS-1832/13 cells are grown in a monolayer with similar oxygenation rates for all cells. Islets are cell clusters and as oxygenation in cultured islets happens only through diffusion from the culture medium, the individual cells will experience a differential re-oxygenation rate after hypoxia.

Previous and present experiments have revealed different susceptibility to damaging effects of hypoxia in human and rat islets. Hence, in previous experiments the exposure to 0.8% hypoxia caused damage to rat islets, including reductions in mitochondrial complexes [18] whereas, as shown here, human islets exposed to 0.8% hypoxia were more resilient, demonstrating up-regulation of mitochondrial complexes comparable to those seen in rat islets exposed to 2.8% oxygen. Such differences underscore the need to test out the degree and duration of hypoxia that lead to pre-conditioning (which could be beneficial in an islet transplantation setting) without causing major and irreversible damage. In general it is recognized that a precarious balance exists between the degree and duration of hypoxia which causes adaptation and that which causes irreversible damage [30].

The overnight lasting effects of hypoxia were moderate, particularly in the intact cells. From a physiological point of view this could perhaps be expected, as mitochondrial function may only vary so much without compromising viability. In that context it is interesting that up-regulation of mitochondrial complexes after previous hypoxia were possibly more marked than respirometric effects. This could indicate that individual components of mitochondria of β-cells have considerable and rapidly recruitable power of adaptation.

## Conclusion

Our results provide evidence for hypoxic adaptation in β-cells in the form of increased mitochondrial ATP production as a result of both higher oxidative capacity and more efficient oxidative phosphorylation. Basal respiration did not seem to be affected, indicating, perhaps, that adaptive changes succeed in maintaining the necessary basal ATP production. Indirect evidence for at least partial success of adaptation to retain functionality is the preservation of glucose induced insulin secretion that could be demonstrated in the clonal beta cells and in rat and in human islets alike. Evoking adaptive responses could be valuable in optimizing conditions for the success of islet transplantation.

## Supporting Information

S1 Fig A titration protocol for evaluating effects of malate. Shown is sequential titration of malate to a permeabilized cell sample in the presence of ADP (5 mM) and presence or absence of glutamate (G, 10 mM).
(TIF)

S2 Fig Optimization of digitonin concentration. Before addition of digitonin, a stable oxygen flux in the presence of succinate, ADP and rotenone was attained. Digitonin was titrated in steps of 1 μM, with stable oxygen flux observed at every concentration before continued titration.
(TIF)

S1 Text Supporting information to Results, section Effects of hypoxia on respiration in intact INS-1832/13 cells. In a separate series of experiments we employed a shorter period of hypoxia (8 h instead of 18 h) followed by re-oxygenation. Results for respiration capacity (ETS) expressed by DNA contents were: 10.96 ± 0.65 at normoxia vs. 11.96 ± 0.70 nmol O_2_/s/ng DNA/10^6^ cells at hypoxia (n = 5, P < 0.05).
(PDF)
