# Supplementary figures and images for: Mitochondrial Respiration in Insulin-Producing β-Cells: General Characteristics and Adaptive Effects of Hypoxia
Source: PLoS One. 2015 Sep 24;10(9):e0138558. doi: 10.1371/journal.pone.0138558 (PMC4581632; doi:10.1371/journal.pone.0138558)

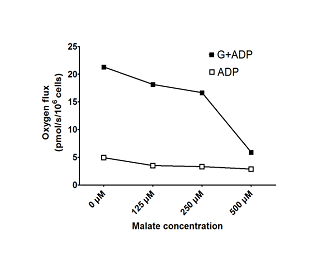

Supplement: S1 Fig — Shown is sequential titration of malate to a permeabilized cell sample in the presence of ADP (5 mM) and presence or absence of glutamate (G, 10 mM). (TIF) [file pone.0138558.s001.tif]

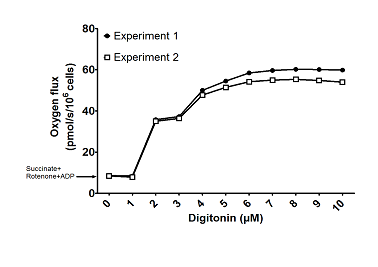

Supplement: S2 Fig — Before addition of digitonin, a stable oxygen flux in the presence of succinate, ADP and rotenone was attained. Digitonin was titrated in steps of 1 μM, with stable oxygen flux observed at every concentration before continued titration. (TIF) [file pone.0138558.s002.tif]
