# Supplementary material for: Mitochondrial Respiration in Insulin-Producing β-Cells: General Characteristics and Adaptive Effects of Hypoxia
Source: PLoS One. 2015 Sep 24;10(9):e0138558. doi: 10.1371/journal.pone.0138558 (PMC4581632; doi:10.1371/journal.pone.0138558)
Supplement: S1 Text — In a separate series of experiments we employed a shorter period of hypoxia (8 h instead of 18 h) followed by re-oxygenation. Results for respiration capacity (ETS) expressed by DNA contents were: 10.96 ± 0.65 at normoxia vs. 11.96 ± 0.70 nmol O2/s/ng DNA/106 cells at hypoxia (n = 5, P < 0.05). (PDF) [file pone.0138558.s003.pdf]

Supporting Information

S1 Text. Supporting information to Results, section **Effects of hypoxia on respiration in intact INS-1832/13 cells.**

In a separate series of experiments we employed a shorter period of hypoxia (8 h instead of 18 h) followed by re-oxygenation. Results for respiration capacity (ETS) expressed by DNA contents were: 10.96 ± 0.65 at normoxia vs. 11.96 ± 0.70 nmol O_2/s/ng DNA/10^6 cells at hypoxia ($n = 5$, $P < 0.05$).
